# Risk factors for postoperative stroke in adults patients with moyamoya disease: a systematic review with meta-analysis

**DOI:** 10.1186/s12883-019-1327-1

**Published:** 2019-05-15

**Authors:** Wei Wei, Xin Chen, Jun Yu, Xu-Qin Li

**Affiliations:** 1 Department of Neurosurgery, Affiliated Dalian Municipal Central Hospital, Dalian Medical University, Dalian, 116033 China; 2 Department of Epidemiology, School of Public Health, Dalian Medical University, Dalian, 116044 China

**Keywords:** Moyamoya disease, Postoperative complication, Risk factors, Meta-analylsis

## Abstract

**Background:**

This systematic review and meta-analysis aimed to clarify the risk factors for postoperative stroke in adult patients with moyamoya disease (MMD).

**Methods:**

We comprehensively searched MEDLINE/PubMed, Web of Science, and Cochrane Library for eligible published literature with regard to the risk factors and postoperative complications in adult patients with MMD. Statistical analysis was conducted using Stata version 12.0. Pooled odds ratio (OR) with 95% confidence interval (CI) were assessed for each risk factor.

**Results:**

There were 8 studies encompassing 1649 patients who underwent surgery with MMD were selected for analysis. Preoperative ischemic event significantly increase the risk of postoperative stroke events (OR = 1.40; 95%CI = 1.02–1.92; *P* = 0.039). PCA involvement correlate with an increased risk of post-infarction (OR = 4.60; 95%CI = 2.61–8.11; *P* = 0.000). Compared to direct bypass, patients who underwent indirect bypass or combined bypass could significantly increase the risk of postoperative stroke events. (OR = 1.17; 95%CI = 1.03–1.33; *p* = 0.017). MMD patients with diabetes were associated with an increased risk of postoperative stroke events (OR = 4.02, 95% CI = 1.59–10.16; *p* = 0.003). MMD patients with hypertension, age at onset and male sex were not associated with an increased risk of postoperative stroke events (*P* > 0.05).

**Conclusions:**

This systematic review and meta-analysis indicated that preoperative ischemic events, PCA involvement and diabetes were independent risk factors for postoperative stroke in MMD patients. Therefore, in order to ensure the curative effect of patients with MMD, it is very necessary to detect these risk factors and prevent postoperative complications in time.

## Introduction

Moyamoya disease (MMD) is an abnormal cerebrovascular characterized by progressive stenosis or occlusion of the intracranial vessels, resulting in the formation of a fine vascular network (the “moyamoya” vessels) at the base of the brain [1]. The current concept is to prevent cerebral ischemia and avoid cerebral hemorrhage [2]. And revascularization surgery is the most effective treatment for MMD. Surgical revascularization can be mainly divided into indirect bypass, direct bypass and combined bypass [3]. However, the incidence of postoperative complications, such as postoperative ischemic or hemorrhagic events and cerebral hyperperfusion syndrome (CHS), has been increasingly reported. These procedure-related complications can seriously affect the prognosis [4].

Previous studies have attempted to determine risk factors that would predict postoperative complications [5–12]. To identify the clinical factors can be more useful in preventing postoperative complications. And, this information regarding the management of adult onset MMD is important [13]. However, the risk factors of postoperative complications were still unclear. Therefore, it is particularly important to identify these risk factors so as to ensure the efficacy of surgical treatment. Thus we conducted this systematic review with meta-analysis to clarify the risk factors for postoperative stroke in adult patients with MMD.

## Methods

This systematic review was performed according to the Preferred Reporting Items for Systematic Reviews and Meta-Analyses statement (PRISMA) criteria [14]. Ethical approval is not required by our institution for secondary research using published scientific studies.

### Data sources and searches

We comprehensively searched MEDLINE/PubMed, Web of Science, and Cochrane Library for eligible published literature with regard to the risk factors and postoperative complications in adult patients with MMD. The relevant studies were included until Dec 2018. The key words used in the search included “moyamoya disease”, “risk factors”, “revascularization”, “postoperative complications “postoperative ischemic” and “hemorrhagic events”. We also manually searched the reference lists of all accepted papers so that no studies were overlooked.

### Study selection

Initially, we identified all possible preoperative risk factors of postoperative complications on univariate and multivariate analysis. We then restricted the systematic review to seven preoperative risk factors, which were the most consistent and amenable to analysis: male sex, age at onset, preoperative ischemic events, past medical history, posterior cerebral artery (PCA) involvement, Suzuki stage [1], and surgical type.

Two investigators independently reviewed abstracts and full-text articles against inclusion and exclusion criteria. Disagreements were resolved through discussion or consultation with a third investigator. Inclusion criteria were as follows: 1) Postoperative complications are related to ischemic or hemorrhage events; 2) Study design was retrospective or prospective observational study; 3) Studies reported odds ratio (OR) or hazard ratio (HR) with 95% confidence interval (CI); 4) Treatment of patients must be surgery; 5) Quality score > 5. Studies were excluded if they were not clinical study; if postoperative complications were not ischemia events or intracranial hemorrhage.

### Data extraction and quality assessment

One investigator abstracted data from the included studies, and a second investigator checked data for accuracy. We abstracted study design detail, patients’ characteristics, adverse postoperative events and preoperative possible risk factors.

Preoperative ischemic event included transient ischemic attacks (TIAs) and infarction. And in our study postoperative complications included hemorrhage events and/or ischemic events, which were not separate statistics in some research. Therefore, to unify the studies, we defined stroke as a new neurologic deficit with radiographic correlation, including ischemic stroke (IS) and hemorrhagic stroke (HS). And past medical history in this study mainly contained hypertension and diabetes. Surgery types were categorized into direct bypass (DB), indirect bypass (IB) and combined bypass (CB). The direct bypass meant superficial temporal artery to middle cerebral artery (STA-MCA) anastomosis. The indirect bypass included encephalo-myo-synangiosis (EMS), encephaloduroarteriosynangiosis (EDAS) and pial synangiosis. And the combined bypass included direct bypass and indirect bypass. PCA involvement and Suzuki stage were confirmed with preoperative digital subtraction angiography (DSA) and/or MR angiography (MRA).

Two investigators independently assessed the quality of the included studies by using the Newcastle–Ottawa Scale (NOS) [15]. The NOS allocates a maximum of 9 points to each of 3 categories: 1) patient selection (3 items), 2) comparability of the 2 study arms (2 items), and 3) assessment of outcome (2 items). Each studies was assigned a final quality rating of good (7–9 points), fair (5–6 points), or poor (0–4 points). Disagreements among investigators were resolved through discussion or consultation with a third investigator. We excluded studies as poor quality.

### Statistical analysis

Statistical analysis was conducted using Stata version 12.0 (Stata Corp). OR or HR with 95%CI were assessed for each risk factor. Heterogeneity of the studies was measured using *I*^*2*^ statistic. Adjusted OR from multivariate analyses were preferred, when multivariate analyses were not reported, the OR from univariate analyses was used. When *I*^*2*^>50% or *P* value<0.05 was identified for heterogeneity among studies, we used the random effect model; Otherwise, a fixed effects model was adopted. Potential publication bias examined by Begg’s rank correlation test and sensitivity analysis was also conducted. All significance testing was 2-sided, and the results were considered statistically significant at *P* < 0.05.

## Results

**Fig. 1:**
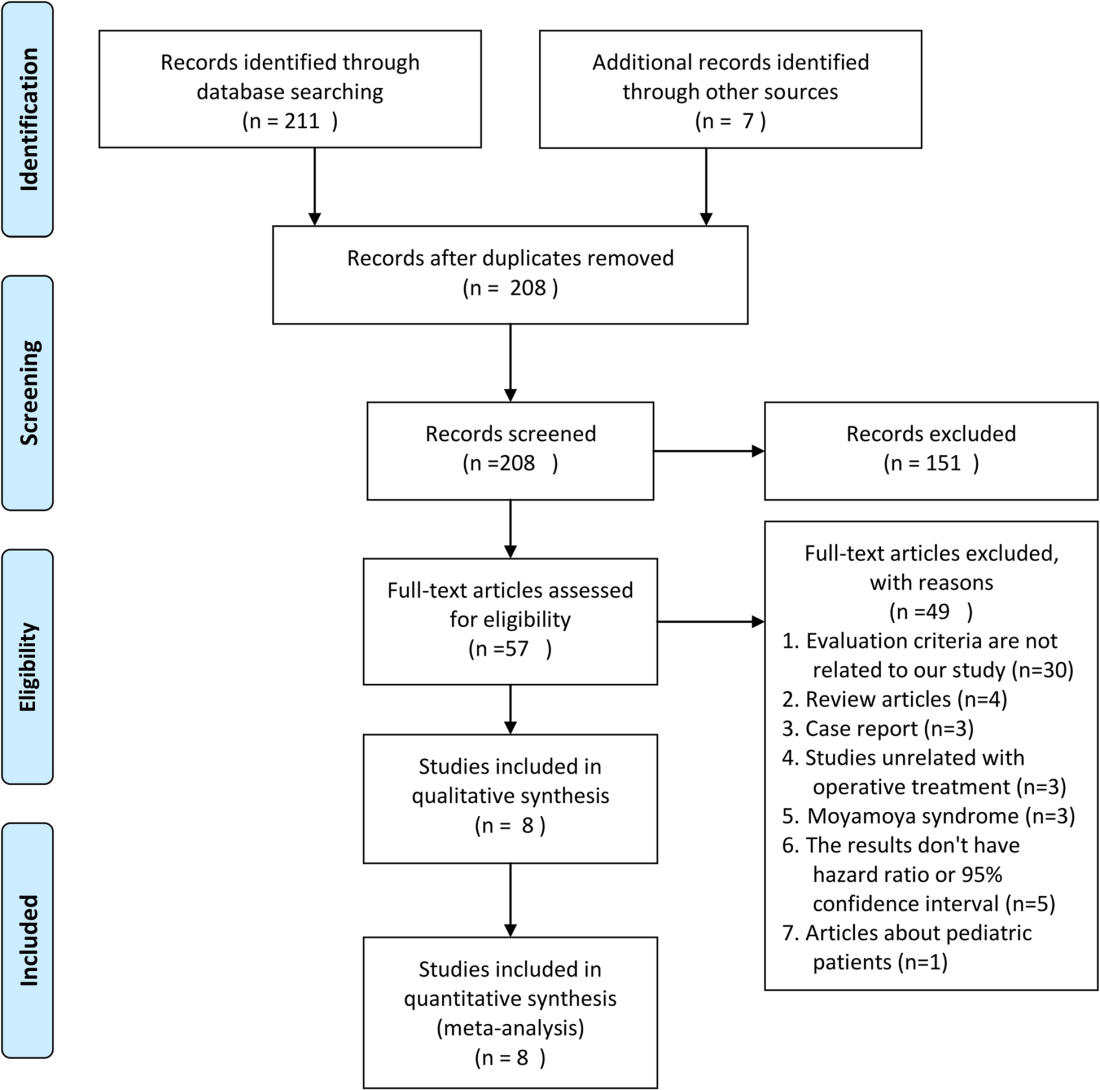
Flow chart of study selection

There were 8 studies [5–12] encompassing 1649 patients who underwent surgery with MMD were selected for analysis. The detailed study selection progress was shown in Fig. 1. The main characteristics of included studies are summarized in Table 1. The included studies were consisted of 3 prospective studies [6, 11, 12] and 5 retrospective studies [5, 7–10].

**Table 1:** Characteristics of included studies

Study	Race	Number of surgery patients	Age (mean ± sd., or range)	Type of study	Onset symptoms	Adverse postoperative events (number)	Preoperative possible risk factors	Quality
Meng 2018 [5]	Asian	500	37.5 ± 9.4	Retrospective	IS; HS; NS	IS (62)	age, male sex, ischemic presentation, hypertension, diabetes, Suzuki stage, PCA involvement, surgery type	High
Peicong 2017 [7]	Asian	87	54.0 ± 3.7	Retrospective	IS; HS	IS (5) + HS (1)	diabetes, PCA involvement	High
Meng 2017 [6]	Asian	121	35.4 ± 7.5	Prospective	IS; NS	IS (7) + HS (2)	age, male sex, diabetes, hypertension, PCA involvement, Suzuki stage, surgery type	High
Wonhyoung 2016 [8]	Asian	194	37.2 ± 11.4	Retrospective	IS; HS; NS	IS (43)	Infarction, TIA, PCA involvement, surgery type	High
Antonucci 2015 [9]	Caucasian	31	18–67	Retrospective	IS; NS	IS (8)	Infarction	High
Tackeun 2015 [10]	Asian	201	38.3 ± 11.6	Retrospective	IS; NS	IS (4) + HS (1)	age, male sex, Suzuki stage	Fair
Gross 2013 [11]	Caucasian	45	39.2 ± 12.2	Prospective	IS; HS; NS	IS (5) + HS (1)	age, Infarction, surgery type	High
Xiangyang 2012 [12]	Asian	470	36.8 (18–59)	Prospective	IS; HS; NS	IS (43) + HS (17)	age TIA, PCA involvement, male sex, infarction, Suzuki stage	High

*IS* ischemic stroke, *HS* hemorrhagic stroke, *NS* nonspecific symptom, *sd.* standard deviation

### Ischemic event

**Fig. 2:**
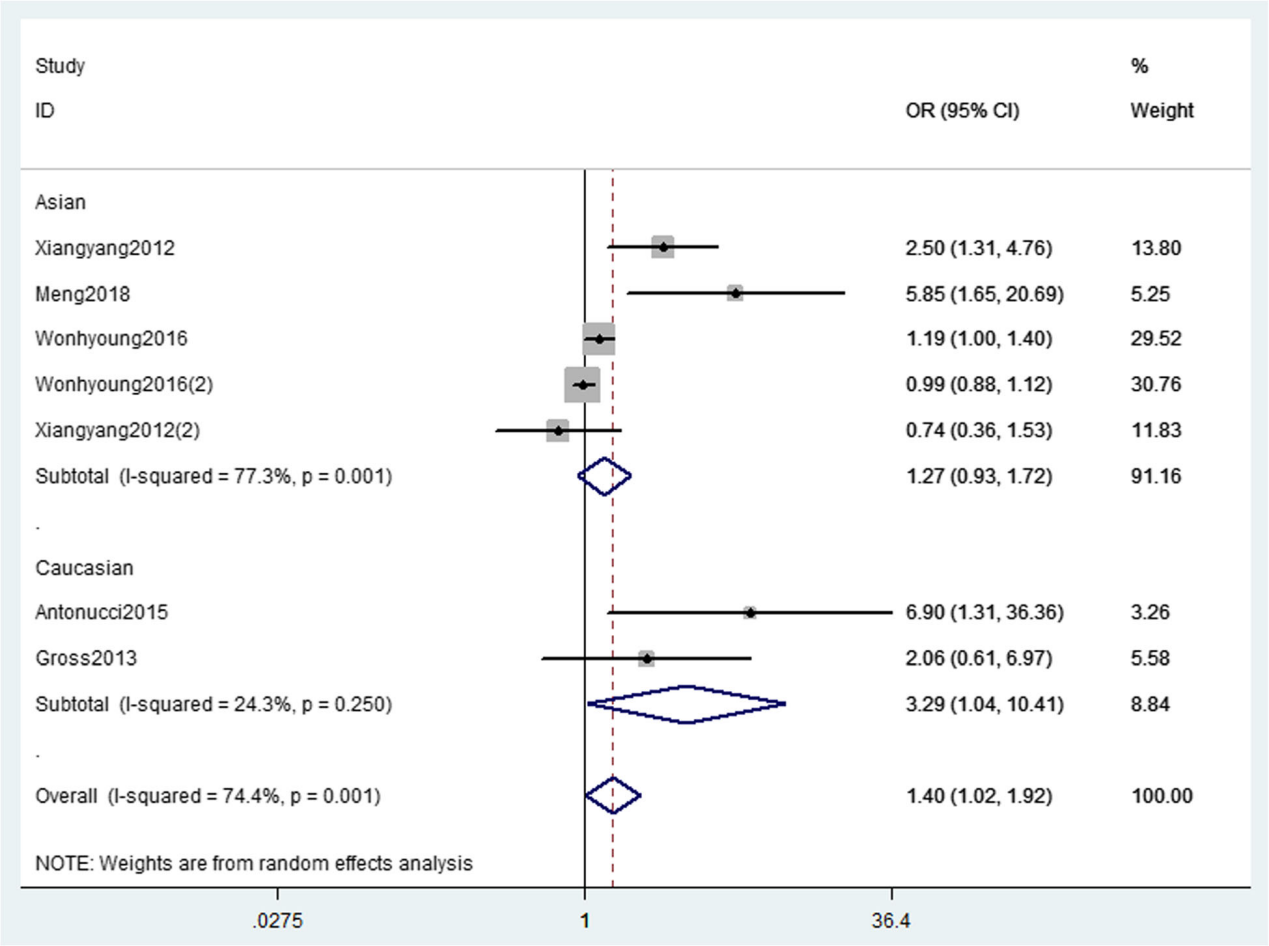
The forest plot of association between preoperative ischemic event and postoperative stroke events

Five studies [5, 8, 9, 11, 12] with 1240 patients about preoperative ischemic event were included in the pooled analysis (Fig. 2). Among them, two studies [8, 12] reported results of TIAs and infarction, respectively. Heterogeneity across studies was detected (*I*^2^ = 74.4%; *p* = 0.001), so random-effect model was applied. In this analysis, preoperative ischemic event significantly increase the risk of postoperative stroke events (OR = 1.40; 95%CI = 1.02–1.92; *P* = 0.039). The results of subgroup analyses were stratified by race, MMD patients in Caucasian with preoperative ischemic event had a significant increase in risk for post-stroke (OR = 3.29, 95%CI = 1.04–10.41; *P* = 0.043), whereas the pooled OR revealed a non-significant increase in Asian (OR = 1.27; 95%CI = 0.93–1.72; *P* = 0.130).

### PCA involvement

**Fig. 3:**
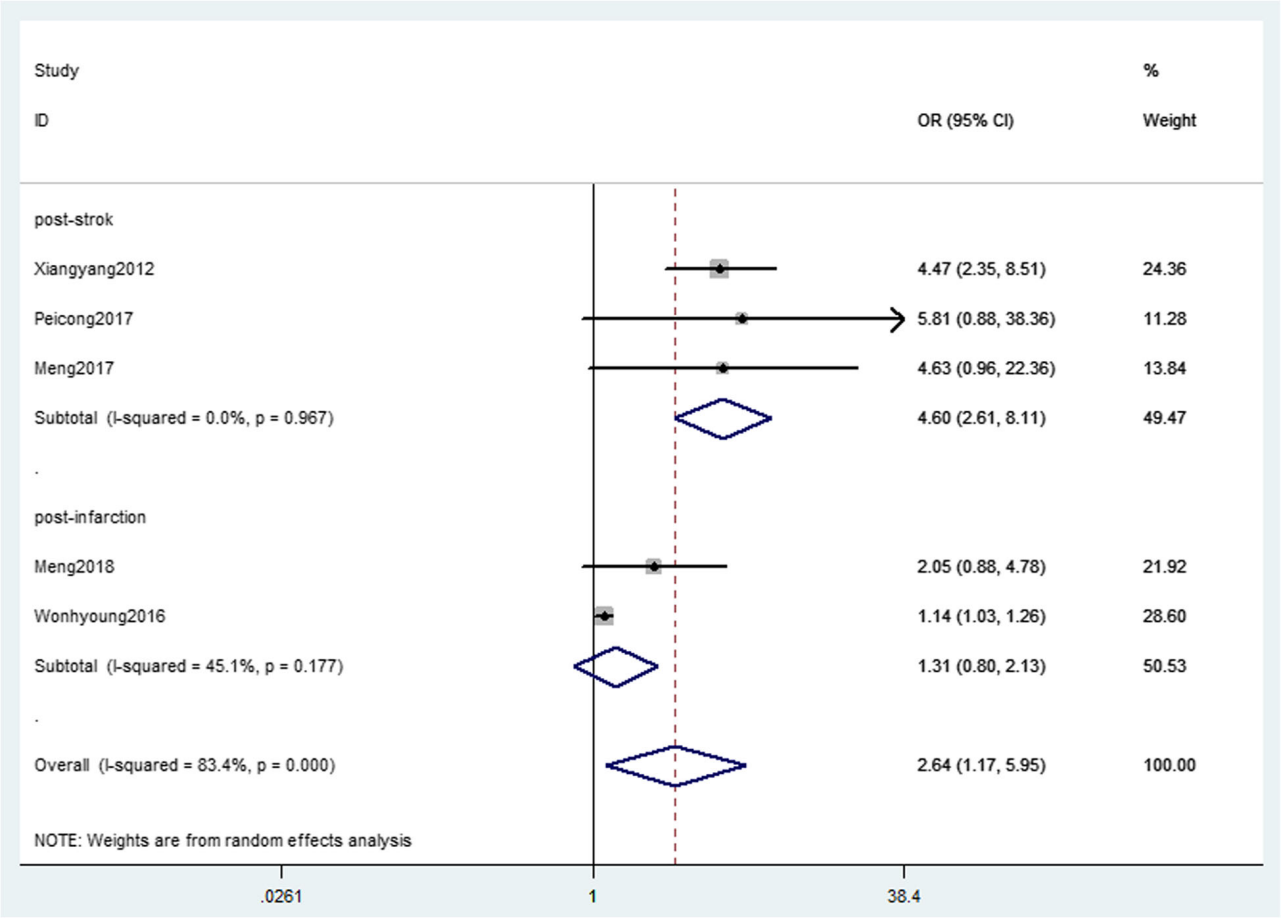
The forest plot of association between PCA involvement and postoperative stroke events

Five studies [5–8, 12] with 1372 patients about PCA involvement were pooled in the analysis (Fig. 3). Heterogeneity across studies was detected (*I*^2^ = 83.4%; *p* = 0.000), so random-effect model was applied. Patients with PCA involvement had a strong significant increase in risk for postoperative stroke events (OR = 2.64; 95%CI = 1.17–5.95; *p* = 0.019). In subgroup analysis, PCA involvement correlate with an increased risk of post-infarction (OR = 4.60; 95%CI = 2.61–8.11; *P* = 0.000), whereas there is no association between PCA involvement and risk of in post-stroke (OR = 1.31; 95%CI = 0.80–2.13; *P* = 0.280).

### Surgery types

Among 4 studies [5, 6, 8, 11], 454 patients underwent DB, 295 patients underwent IB, and 265 patients underwent CB. No heterogeneity across studies was detected (*I*^2^ = 0.00%; *p* = 0.601), so fixed-effect model was applied. Compared to DB, patients who underwent IB or CB could significantly increase the risk of postoperative stroke events. (OR = 1.17; 95%CI = 1.03–1.33; *p* = 0.017). The study by Wonhyoung et al. [8] contributed importantly to the pooled OR (weight 96.08%), when this study was omitted in the model, the results was statistically insignificant (OR = 1.31; 95%CI = 0.69–2.49).

### Suzuki stage

For studies [5, 6, 10, 12] with 1292 patients contributed to pooled outcome. Moderate heterogeneity across studies was detected (*I*^2^ = 32.8%; *p* = 0.215), fixed-effect model was applied. Preoperative Suzuki stage was not associated with the risk of postoperative stroke events (OR = 1.20; 95%CI = 0.97–1.49; *p* = 0.101). The study by Xiangyang et al. [12] contributed importantly to the pooled OR (weight 71.65%), when this study was omitted in the model, the results was statistically significant (OR = 1.70; 95%CI = 1.13–2.57).

### Age at onset

Five studies [5, 6, 10–12] with 1292 patients contributed to analysis. Moderate heterogeneity across studies was detected (*I*^2^ = 38.2%; *p* = 0.167), fixed-effect model was applied. In MMD patients, age at onset were marginally associated with an increased risk of postoperative stroke events (OR = 1.02; 95% CI = 1.00–1.04; *p* = 0.090).

### Male sex

Four studies [5, 6, 10, 12] with 1292 patients contributed to pooled outcome. No heterogeneity across studies was detected (*I*^2^ = 0.00%; *p* = 0.726), so fixed-effect model was applied. In MMD patients, male sex was not associated with an increased risk of postoperative stroke events (OR = 1.16, 95%CI = 0.75–1.82; *p* = 0.504). The study by Xiangyang et al. [12] contributed importantly to the pooled OR (weight 52.74%), but when this study was omitted in the model, it remained statistically insignificant.

### Medical history

Three studies [5–7] with 587 patients and two studies [5, 6] with 621 patients respectively reported the association between MMD patients with diabetes and hypertension and postoperative stroke events. No heterogeneity across studies was detected (*I*^2^ = 5.90%, *p* = 0.345; *I*^2^ = 0.00%, *p* = 0.494, respectively), so fixed-effect model was applied. MMD patients with diabetes were associated with an increased risk of postoperative stroke events (OR = 4.02, 95% CI = 1.59–10.16; *p* = 0.003). However, MMD patients with hypertension were not associated with an increased risk of postoperative stroke events (OR = 0.70, 95% CI = 0.31–1.58; *p* = 0.392).

### Publication bias

The Begg’s rank correlation test indicated no evidence of publication bias among the included studies regarding the risk of male sex, age at onset, preoperative ischemic events, PCA involvement, medical history, Suzuki stage and surgical type (*P* > 0.05) (Table 2).

**Table 2:** Results of pooled OR with 95%CI and Begg’s test for preoperative possible risk factors

Preoperative possible risk factors	Number of articles	*I* ^2^	*P* value for heterogeneity	Pooled OR(95%CI)	*P* for pooled results	*P* for Begg’s test
Ischemic events	5	74.4%	0.001^*^	1.40 (1.02–1.92)	0.039^*^	0.230
PCA involvement	5	83.4%	0.000^*^	2.64 (1.17–5.95)	0.019^*^	0.806
Suzuki stage	4	32.8%	0.215	1.20 (0.97, 1.49)	0.101	0.734
Surgery type (DB as reference)	4	0.0%	0.601	1.12 (1.03, 1.33)	0.017^*^	0.806
Age	5	38.2%	0.167	1.02 (1.00, 1.04)	0.090	0.806
Mae sex	4	0.0%	0.595	1.16 (0.75–1.82)	0.504	1.000
Diabetes	3	5.9%	0.345	4.02 (1.59, 10.16)	0.003^*^	0.296
Hypertension	2	0.0%	0.494	0.70 (0.31, 1.58)	0.392	–

*DB* direct bypass

^*^p < 0.05

## Discussion

MMD is a chronic cerebrovascular disorder. With recent advances in neuroradiological diagnostic modalities, the diagnosis of adult onset MMD has become more frequent than in the past. Postoperative stroke was common complication in patients with MMD. The previously reported rate of postoperative ischemia in MMD patients after revascularization varies from 1.5 to 11.4% [16–19]. Some preoperative factors have been previously reported to be associated with increase of postoperative ischemic complications in MMD patients [5–12, 16, 20–25]. Controversy still remains in the literature regarding which contribute as risk factors.

In this meta-analysis, preoperative ischemic event, PCA involvement, presence of diabetes and surgical type of IB and CB were verified to be independent risk factors associated with postoperative stroke. Preoperative ischemic event was likely to be an indicator of the instability of cerebral hemodynamics before surgical revascularization [8]. Previous studies had reported that frequent occurrence of preoperative TIAs was an important indicator of a risk for perioperative ischemic [22] and stroke [26] complications. Our results showed that preoperative ischemic event had a significant increase in risk for postoperative stroke. Interestingly, in the subgroup analysis, this association only exit in Caucasian, but not in Asian. That may be due to our study results included not only TIAs but also symptomatic infarction. Our findings suggested that PCA involvement was an independent risk factor, possibly because patients with advanced stage MMD, the leptomeningeal collateral from the PCA was significantly important collateral blood flow source [8]. Thus, PCA impaired may seriously affect cerebral hemodynamics, which may lead to postoperative cerebral ischemia. Besides, Jung et al. [17] reported that direct bypass may result in cerebral infarction of contralateral hemisphere in patients with PCA involvement. In the past medical history of patients, several previous reports suggested that diabetes as a predictor of recurrent stroke [27–29]. A recent meta-analysis reported that diabetes is an independent risk factor for stroke recurrence [30]. In context of MMD, patients with diabetes may elevate expression of growth factors and cytokines, such as hepatocyte growth factor, transforming growth factor-β, vascular endothelial growth factor, and nitrotyrosine, which could lead to more collateral angiogenesis [31]. Though our results support this hypothesis, it should be noted that only three included studies considered diabetes as a risk factor. For surgery types, a recent meta-analysis proves that direct bypass could reduce the risk of perioperative stroke than indirect bypass in MMD [32]. Notably, we also found that the risk of postoperative stroke is higher in MMD patients who underwent indirect bypass and combined bypass compared with direct bypass. However, due to sensitivity analysis unstable (the result become insignificant when excluded Wonhyoung et al. [8]), that conclusion should be interpreted cautiously.

Patients with higher Suzuki stage probably had poor collateralization pathways to compensate for the hemodynamic impairment [5]. However, in the current study, sensitivity analysis of the association between Suzuki stage and recurrent stroke was instable, the result become significant after exclude the Xiangyang et al. [12] We also found that older age of symptom onset was identified as a possible predictor of postoperative strokes. This may be because older patients are often accompanied by some underlying diseases, such as diabetes, arteriosclerosis, and hypertension and so on, these may aggravate postoperative complications. Therefore, further studies with a large sample size are still needed to confirm these factors.

There are some limitations in this meta-analysis. First, our research included three prospective studies, which reported HR value of different preoperative risk factors. Thus, pooled HR value of risk factors for postoperative stroke could not be obtained. Second, among different ethnic groups, there may be differences in postoperative stroke [33], so racial differences may exist. However, in the current study, there is not enough data to explore the racial differences in other risk factors. Third, heterogeneity exists in the meta-analysis results of ischemic events and PCA involvement, it could be inter-institutional differences, such as clinical experience, diagnostic standard, operative techniques, and surgeon’s preferences, however, we did not find the cause of heterogeneity. Fourth, due to the limitation of data included in the studies, we only compared between DB and IB/CB (DB as a reference), so further research is still needed to determine the results with IB and CB. In addition, hyperperfusion syndrome was barely mentioned in the included studies, so it is impossible to analyze the influence of factors on hyperperfusion syndrome. Lastly, sensitivity analysis of surgery types and Suzuki stage are instable. Wonhyoung et al. [8] and Xiangyang et al. [12] have a serious impact on the results, respectively.

## Conclusions

This systematic review and meta-analysis indicated that preoperative ischemic events, PCA involvement and diabetes were independent risk factors for postoperative stroke in MMD patients. Therefore, in order to ensure the curative effect of patients with MMD, it is very necessary to detect these risk factors and prevent postoperative complications in time.

## Abbreviations

CB: Combined bypass
CHS: Cerebral hyperperfusion syndrome
DB: Direct bypass
HS: Hemorrhagic stroke
IB: Indirect bypass
IS: Ischemic stroke
MMD: Moyamoya disease
PCA: Posterior cerebral artery
TIAs: Transient ischemic attacks
